# Vascular risk profile and changes of arterial hypertension after surgical revascularization in adult Moyamoya patients

**DOI:** 10.1038/s41598-024-61966-8

**Published:** 2024-05-29

**Authors:** Patrick Haas, Lucas Moritz Wiggenhauser, Jonas Tellermann, Helene Hurth, Daniel Feucht, Marcos Tatagiba, Nadia Khan, Constantin Roder

**Affiliations:** 1 Department of Neurosurgery and Moyamoya Center, University of Tübingen, Hoppe-Seyler-Straße 3, 72076 Tübingen, Germany; 2 Moyamoya Center, University Children’s Hospital of Zurich, University of Zurich, Steinwiesstrasse 75, 8032 Zurich, Switzerland

**Keywords:** Cerebral revascularization, Moyamoya, Arterial hypertension, Comorbidities, Cerebrovascular disorders, Neurovascular disorders, Stroke

## Abstract

Moyamoya disease (MMD) is a rare stenoocclusive cerebral vasculopathy often treated by neurosurgical revascularization using extracranial-intracranial bypasses to prevent ischemic or hemorrhagic events. Little is known about the vascular risk profile of adult MMD patients compared to the general population. We therefore analyzed 133 adult MMD patients and compared them with data from more than 22,000 patients from the German Health Update database. Patients with MMD showed an age- and sex-adjusted increased prevalence of arterial hypertension, especially in women between 30 and 44 years and in patients of both sexes between 45 and 64 years. Diabetes mellitus was diagnosed significantly more frequently in MMD patients with increasing age, whereas the vascular risk profile in terms of obesity, nicotine and alcohol consumption was similar to that of the general population. Antihypertensive medication was changed one year after surgical revascularization in 67.5% of patients with a tendency towards dose reduction in 43.2% of all patients. After revascularization, physicians need to be aware of a high likelihood of changes in arterial hypertension and should adjust all other modifiable systemic vascular risk factors to achieve the best treatment possible.

## Introduction

Moyamoya disease (MMD) is a steno-occlusive arteriopathy primarily affecting the terminal segments of the distal internal carotid artery and its proximal branches. The etiology is not yet clear, predisposing genetic and environmental factors are discussed^1^. Clinically, MMD becomes symptomatic by acute ischemic or hemorrhagic events, or chronic changes such as cerebral atrophy^2^. The patient population is mostly divided by two age peaks in child- and young adulthood^3,4^. In contrast to the much more common systemic atherosclerotic vascular disease, the vascular risk profile of MMD has been poorly examined and understood to date. It is our hypothesis that patients with MMD might often suffer from arterial hypertension (AHT), whereas it is unclear if this is compensatory to increase the cerebral blood-flow, or if the patients may exhibit a different vascular risk profile than the general population. Therefore, we aimed to analyze the vascular risk profile and possible changes of blood pressure after surgical revascularization in our MMD cohort compared to data of national health monitoring in the general German population. Special attention was paid to modifiable vascular risk factors such as obesity, diabetes mellitus (DM) and AHT, as the latter should be considered in the management of MMD due to its direct influence on cerebral perfusion pressure (CPP). In addition, an analysis of changes of the number and/or dose of antihypertensive medication one year after completed surgical revascularization was performed.

## Methods

### Inclusion criteria and data collection

All adult MMD patients ≥ 18 years of age treated at our Moyamoya Center with an inpatient stay between 11/2012 and 02/2023 were included in this retrospective cohort analysis. Diagnosis and disease classification were according to the 2021 Japanese guidelines for Moyamoya disease^5^. Diagnosis was confirmed by conventional cerebral angiography in all cases. If antihypertensive drugs (AHD) were taken, this was recorded as arterial hypertension requiring medication (mAHT). If no AHD was taken, the noninvasive blood pressure readings recorded multiple times on the day of admission were averaged and divided into the appropriate blood pressure categories according to European Society of Cardiology/European Society of Hypertension (ESC/ESH) Guideline criteria^6^. If AHT grade ≥ 1 or isolated systolic hypertension (ISH) was present, this was considered as undetected AHT (uAHT). DM, nicotine (NC) and alcohol consumption (AC) were taken from the standardized medical history protocol. Body mass index (BMI) was calculated according to the recording of weight and height. Severity of MMD was classified angiographically according to the Suzuki grading for each hemisphere^7^.

### General population

Data on the prevalence of AHT or DM in the control group were taken from the German Health Update 2014/2015 (GEDA 2014/2015-EHIS) with 23,967 and 23,345 adult participants, respectively^8,9^. Data on BMI and degree of obesity and NC use were taken from the German Health Update 2019/2020 (GEDA 2019/2020-EHIS) with 22,414 and 22,699 adult subjects, respectively^10,11^. The comparison with the studied MMA collective was gender- and age-adjusted.

### Antihypertensive drugs

Dosage and number of drug classes of AHD were recorded as surrogate parameters for the change in AHT after revascularization. To ensure comparability in the case of combination therapies or drug changes, the total daily dose per drug was normalized to the approved maximum dose according to the national expert information.

### Data and statistics

REDCap database software (version 9.6.1) was used to store patient data. Data analysis and statistics were performed in JMP (JMP 16.0, SAS Institute) and Excel (Microsoft Corporation). The alpha level was defined as 0.05. Independence of category variables was examined using Chi^2^-tests and Odds Ratios (OR) calculated if appropriate.

### Standard protocol approvals, registrations, and patient consents

All procedures performed in studies involving human participants were in accordance with the ethical standards of the institutional research committee and with the 1964 Helsinki declaration and its later amendments or comparable ethics standards. Approval of the ethics committee Tübingen was obtained (144/2018BO2). Due to the retrospective character of this analysis no specific formal consent of the participating patients was obtained.

### Ethical approval

All procedures performed in studies involving human participants were in accordance with the ethical standards of the institutional research committee and with the 1964 Helsinki declaration and its later amendments or comparable ethics standards. Approval of the ethics committee Tübingen was obtained (144/2018BO2).

## Results

### Baseline characteristics

**Figure 1:**
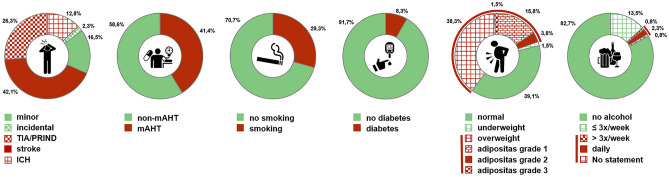
Pie charts showing percentage distribution of prevalence of initial symptoms/manifestations, drug-requiring arterial hypertension, smoking, diabetes, obesity, and alcohol consumption (from left to right). Minor symptoms include headache, dizziness, difficulty concentrating, subjective weakness and loss of performance in daily life and work.

A total of 133 adult MMD patients were included in the analysis. The majority was of Caucasian origin (93.2%) and the female-to-male ratio was 2.6: 1 with a mean age of 41.9 years ± 13.3. Unilateral MMD was present in 46 cases, divided into n = 25 (18.8%) right-sided and n = 21 (15.8%) left-sided cases, whereas 87 patients were affected bilaterally (65.4%). Ischemic strokes (42.1%) and temporary perfusion deficits such as transient ischemic attacks (TIA) or prolonged ischemic neurological deficits (PRIND) (26.3%) were the most common initial clinical manifestations (Fig. 1). Patients without AHT (non-mAHT) exhibited a significantly higher rate of ischemic events (non-mAHT 79.5% vs. mAHT 52.7%; *p* = 0.0011, OR 3.5). Primary intracerebral hemorrhage (ICH) was less common (12.8%) and occurred more frequently in patients with mAHT than non-mAHT (18.2% vs. 9.0%), although this did not reach statistical significance. Patients with uAHT did not have a higher risk for ICH as a primary clinical manifestation than their counterparts with controlled (mAHT) or normotensive blood pressure levels (non-mAHT). Baseline patient characteristics can be found in Table 1 and Fig. 1.

**Table 1:** Patient characteristics.

	n (% total)/mean ± SD
Sex	133
Female	96 (72.2%)
Male	37 (27.8%)
Age [years]	41.9 ± 13.3
Ethnicity	
Caucasian	124 (93.2%)
Asian	9 (6.8%)
Suzuki grade	
Unilateral right	25 (18.8%)
Grade 1	1 (4.0%)
Grade 2	5 (20.0%)
Grade 3	13 (52.0%)
Grade 4	2 (8.0%)
Grade 5	3 (12.0%)
Grade 6	1 (4.0%)
Unilateral left	21 (15.8%)
Grade 1	1 (4.8%)
Grade 2	3 (14.3%)
Grade 3	12 (57.1%)
Grade 4	3 (14.3%)
Grade 5	1 (4.8%)
Grade 6	1 (4.8%)
Bilateral (patients)	87 (65.4%)
Grade 1 (/hemispheres)	11 (6.3%)
Grade 2 (/hemispheres)	20 (11.5%)
Grade 3 (/hemispheres)	38 (21.8%)
Grade 4 (/hemispheres)	42 (24.1%)
Grade 5 (/hemispheres)	46 (26.4%)
Grade 6 (/hemispheres)	17 (9.8%)
Initial symptoms/manifestation	
TIA/PRIND	35 (26.3%)
Stroke	56 (42.1%)
ICH	17 (12.8%)
Minor (headache, performance deficit)	22 (16.5%)
Incidential	3 (2.3%)
Concomitant aneurysms	9 (6.8%)
mAHT	6 (10.9%)
Non mAHT	3 (3.8%)
AHT	76 (57.1%)
mAHT	55 (41.4%)
uAHT	21 (15.8%)
Grade 1 (ESC/ESH)	12 (57.1%)
Grade 2 (ESC/ESH)	9 (42.9%)
ISH (ESC/ESH)	12 (57.1%)
BMI	26.6 ± 4.8
Obesity grade 1	21 (15.8%)
Obesity grade 2	5 (3.7%)
Obesity grade 3	2 (1.5%)
Overweight	51 (38.3%)
Normal weight	52 (39.1%)
Underweight	2 (1.5%)
Drug abuse	
Nicotine	39 (29.3%)
Alcohol (≥ 3x/week)	4 (3.0%)
Diabetic disorder	11 (8.3%)
Age ≥ 45y	11 (15.9%)

### Arterial hypertension

At the time of initial inpatient presentation, 41.4% of patients had a diagnosed mAHT. This group was significantly older than their non-mAHT counterpart (mean 46.6 vs. 38.7 years, *p* = 0.0002). In n = 78 patients with non-mAHT, multiple measurements during the stay at the hospital revealed a suspicion of previously undetected / untreated AHT (uAHT) in 26.9% patients. Of these, 42.9% of patients with uAHT were categorized to the AHT grade 2 category according to ESC/ESH guidelines. A high rate of isolated systolic hypertension (ISH) was found in n = 12 cases (57.1%) of uAHT patients. Considering mAHT and uAHT patients together, the overall prevalence of AHT in the studied population raised from 41.4% (mAHT) to 57.1% (mAHT and uAHT). MMD concomitant aneurysms occurred more frequently in the mAHT than in in the non-mAHT group (10.9% vs. 3.9%), but without statistical significance.

### Comparison with general population

After correction for age and sex, NC and AC in our cohort were similar to those in the normal population, with no significant differences. Adult female MMD patients under 30 years of age were significantly more likely to be overweight (BMI ≥ 25) than the normal female population (*p* = 0.0013). DM did not occur at all in the younger patients of our collective, but this did not lead to a significant difference given the low prevalence in the general population. In contrast, a steep increase in the prevalence of DM (n = 11) in the group of MMD patients older than 45 years was significant, with an increased OR of 2.87 in men (*p* = 0.0303) and 2.76 in women (*p* = 0.0275).

**Figure 2:**
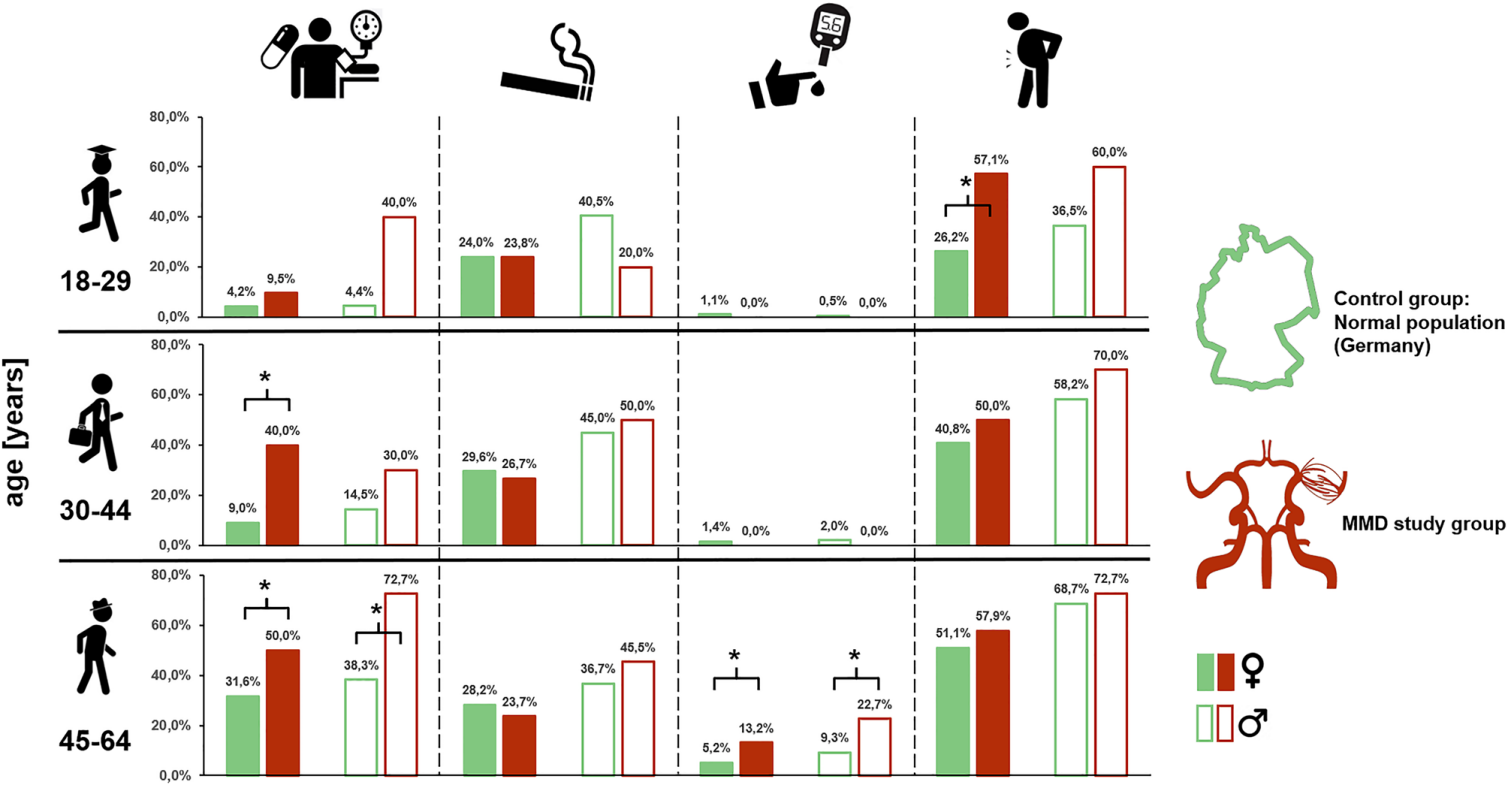
Bar graphs comparing prevalence of drug-requiring arterial hypertension, smoking, diabetes, and obesity (from left to right) between the control group of the German Health Update population (green) and the MMA study population (red), subdivided into the age groups 18–29, 30–44, and 45–64 years, and by sex (filled: female, checkered: male). Asterisks (*) indicate a significant difference with p < .05. Note: the number of male patients with MMD between 18 and 29 years of age was too small to statistically test for significant differences.

Adult MMD patients had a significantly higher prevalence of mAHT than the general population: this was especially true for women between 30 and 44 years (*p* < 0.0001, OR 6.74) and patients between 45 and 64 years (women *p* = 0.0216, OR 2.06; men *p* = 0.0009, OR 4.30) (Fig. 2).

### Changes in arterial hypertension after revascularization

**Figure 3:**
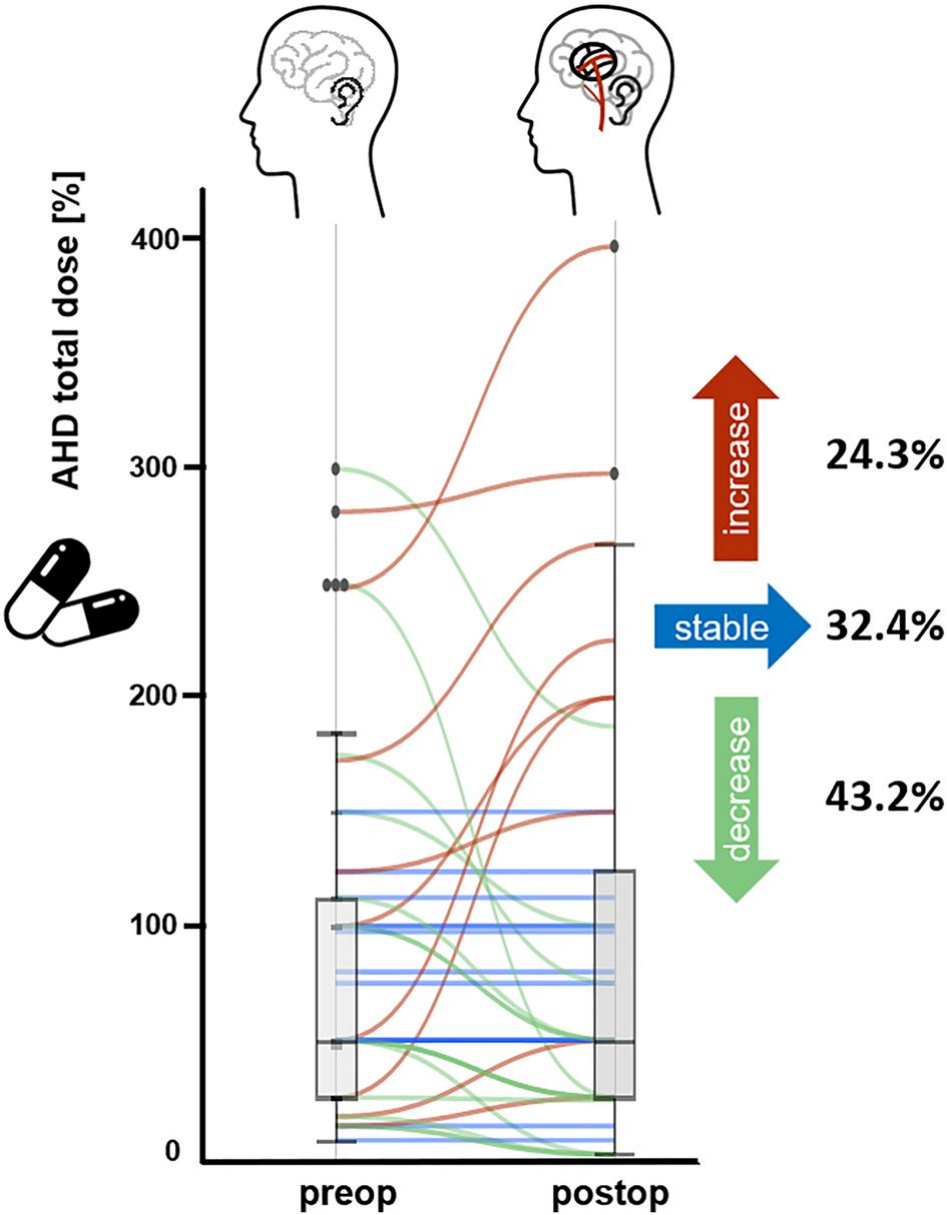
Parallel plot and associated box plots of antihypertensive drugs (AHD) taken pre- and postoperatively (1 year after completed revascularization). Drug dose was recorded as a percentage of the possible maximum dose to allow comparability of different doses of different classes of drugs in monotherapy or polytherapy.

In the studied population, n = 111 patients underwent surgical revascularization, of which n = 80 (72.1%) had a complete inpatient follow-up (FU). In n = 7 patients, further FU took place telemedically, in n = 11 patients 1-year FU is still pending, and n = 13 patients were lost to FU. 67.5% of all patients had a change of AHT medication dose within one year after revascularization. The dose was reduced in 43.2% of all patients, while 32.4% of patients took an unchanged and 24.3% an increased dose (Fig. 3). In 67.6% of all patients with mAHT, the number of medications remained unchanged in the FU, whereas 24.3% of the patients had to take fewer different medications and 8.1% of patients needed to take more.

## Discussion

### Vascular risk profile

In the absence of causal therapies, the management of MMD has focused primarily on revascularization by direct and indirect EC-IC bypasses to prevent from ischemic or hemorrhagic events. In contrast to “classic” stroke of e.g. thromboembolic origin, little attention has been paid to modifiable vascular risk factors in MMD. This might be because their role in the development and progression of the disease, as well as their effect on primary and secondary prevention have been poorly investigated. It is therefore essential to understand whether adult MMD patients have a conspicuous vascular risk profile with regard to the factors identified so far. In smaller case–control studies with 28 and 12 MMD cases, respectively, patients showed a clustered occurrence of DM, AHT, as well as hyperlipidemia compared with non-MMA stroke collectives or healthy controls, comparable to our results^12,13^. In a study of another larger European MMD collective, a comparably high prevalence was reported with respect to DM (11.2%), NC (44.7%), and obesity (24.8%)^3^. Ge et al. prospectively analyzed 138 MMD cases and identified an increased BMI (OR 1.1) and abnormal homocysteine levels (OR 1.2) compared with healthy controls as risk factors for the diagnosis of MMD. Interestingly, in addition to the increased occurrence of DM (11.6%), blood pressure was also elevated in 31.8% compared to controls^14^. Several studies also confirmed an increased association of MMD with DM type 1 and/or 2^15,16^. The influence of a susceptible gene for the development MMD, ring finger protein 213 (RNF213), on insulin regulatory pathways via TNF-alpha-mediated inflammation as a pathomechanism was discussed before^17^. This concurs with our findings showing that with increasing age and independent of sex, the prevalence of DM significantly exceeds that of the normal population. RNF213 also appears to exert an indirect influence on blood flow and thus blood pressure: RNF213 deficient mouse models showed abnormal angiogenesis^18,19^. Secondly, concomitant extracranial arteriopathies possibly occurred more frequently in the RNF213p.Arg4810Lys variant, especially in the homozygous status^20–22^. An autopsy study with evidence of intimal thickening in the pancreas, lung, and also kidney supports the hypothesis of this possible systemic-relevant vascular aspect of MMD^23^. Such intimal thickening leads to renal artery stenosis (RAS), which in pediatric MMD patients is significantly associated with AHT, which itself is less common than in adults, with a prevalence of 5.7–5.9%^24,25^. In adult MMD, RAS is also more common with a prevalence of 2–8%, but still remains a rare cause of arteriopathy-related AHT overall (RAS-AHT)^22,26,27^. Thus, only one patient with RAS-AHT was identified in our study population, although it should be noted that no standard screening tests were performed for this in our cohort (Table 1). Therefore, the frequency of AHT cannot be explained solely by associated systemic arteriopathies alone. Remarkably, we also found a significant difference of AHT compared the normal population, which became apparent even earlier in female than in male MMD patients. With 39.2% in 881 patients, Lu et al. even found a comparably high prevalence of AHT in the Chinese cohort studied^28^. Kraemer et al. found an even higher prevalence of AHT in a European collective, with 50.2% and 54.4% in MMA and MMD, respectively^3^. However, whether AHT is an independent predisposing factor of disease development, part of the disease in terms of systemic arteriopathy, or more likely a compensatory mechanism of decreased cerebral blood flow (CBF) cannot be answered with the present findings alone. At least the latter two points are supported by the observation that AHT developed in 29.0% of cases in long-term FU after revascularization in a South Korean pediatric MMD cohort (n = 131)^29^. Generally spoken, we hypothesize, that AHT is most likely caused as a compensatory mechanism by the brain to improve cerebral hemodynamics in patients with impaired cerebral blood flow.

Interestingly, ischemic events were less frequently the initial manifestation in patients with mAHT of our cohort, so that a drug-controlled blood pressure with target values in the high-normal range might exert a protective effect. This is consistent with the results of a cohort of 542 patients studied, in which severe AHT and the absence of AHD were identified as predictors of poor outcome, whereas the overall prevalence of AHT in MMD was also high (28.8%)^30^. Especially in the light of ischemic events being the most frequent clinical manifestation in European MMD patients, special attention should be paid to blood pressure management perioperatively and during further FU^3,4^. A statistical correlation of AHT with the severity of MMD according to Suzuki grades was not found, nor that the need for AHD changed significantly in number or dosage after completed revascularization. On one hand, this could underline the hypothesis of a systemic arteriopathy and against that of secondarily induced compensatory AHT as an autoregulatory process, since with completed revascularization there should be sufficient CBF and AHT should regress accordingly. On the other hand, it is also conceivable, that the cerebral vascular resistance (CVR) in the precapillary arterioles as a control parameter of CBF is also directly or indirectly affected by MMD, so that a compensatory high-normal blood pressure is still necessary to maintain a sufficient CBF despite placement of bypasses.

Whether there are general advantages or disadvantages of the AHD classes used in MMD has not been systematically investigated so far, but at least for calcium channel blockers (CCB) Han et al. saw a better cerebral perfusion after revascularization^31^. Whether the drug mechanism of CCBs with inhibition of calcium influx into vascular smooth muscle cells might be advantageous in MMD-associated AHT is certainly an interesting question of future research.

### Limitations

According to the retrospective nature of the study, there is always a risk of selection bias. Nevertheless, this is a sufficiently large study cohort to achieve statistical power. In addition, we did not include laboratory parameters due to insufficient data. Dyslipidemia has also been identified as a predisposing factor for cerebrovascular events in asymptomatic MMA patients. Dyslipidemia markers such as cholesterol and the LDL/HDL quotient, but also homocysteine should be considered in further studies. Furthermore, the effect of white coat syndrome (WCS) on the measured blood pressure values should not be underestimated. However, this is primarily relevant for uAHT, which is why mAHT was primarily used for the above analysis. To further confirm the diagnosis of uAHT, further diagnostics such as home blood pressure monitoring are necessary. The effects of WCS in particular also make the interpretation of blood pressure values in the present work difficult, which is why AHD was deliberately chosen as a more reliable primary parameter of blood pressure control. Further, measured blood pressure values are influenced by the respective AHD with a possibly changed dose, making blood pressure values alone (under ongoing medication therapy) useless.

## Conclusion

This case–control study enabled the assessment of a vascular risk profile for MMD patients and to compare it with that of the general population, as well as to quantify the changes in antihypertensive medication after revascularization. The prevalence of AHT in the MMD cohort was significantly higher than in the German population, especially in women aged 30 to 64 and men aged 45 to 64. MMD patients older than 45 were significantly more likely to suffer from DM. The frequencies of other vascular risk factors such as obesity, NC, and AC were comparable to the general population. 1 year after completion of revascularization, a relevant change in AHD was observed in about 2 out of 3 patients. A dose reduction was seen in approximately 43% and a dose increase in 24%. Treating physicians need to be aware of the vascular risk profile of MMD patients with an increased prevalence of DM and AHT and consider adjusting antihypertensive medication after revascularization.

## Data Availability

The datasets generated during and/or analyzed during this study are available from the corresponding author on reasonable request.

## Abbreviations

AC: Alcohol consumption
AHD: Antihypertensive drugs
AHT: Arterial hypertension
BMI: Body mass index
CBF: Cerebral blood flow
CCB: Calcium channel blocker
CPP: Cerebral perfusion pressure
CVR: Cerebral vascular resistance
DM: Diabetes mellitus
ESC: European Society of Cardiology
ESH: European Society of Hypertension
FU: Follow-up
GEDA: German Health Update
ICH: Intracerebral hemorrhage
ISH: Isolated systolic hypertension
mAHT: Arterial hypertension requiring medication
MMD: Moyamoya disease
RAS: Renal artery stenosis
NC: Nicotine consumption
OR: Odds ratio
PRIND: Prolonged ischemic neurological deficits
TIA: Transient ischemic attacks
uAHT: Undetected arterial hypertension
WCS: White coat syndrome
